# Whitening toothpaste containing activated charcoal, blue covarine, hydrogen peroxide or microbeads: which one is the most effective?

**DOI:** 10.1590/1678-7757-2018-0051

**Published:** 2019-01-14

**Authors:** Vanessa Torraca Peraro Vaz, Dandara Proba Jubilato, Morgana Regina Mendonça de Oliveira, Janaina Freitas Bortolatto, Michael Christopher Floros, Andrea Abi Rached Dantas, Osmir Batista de Oliveira

**Affiliations:** 1 Universidade Estadual Paulista (UNESP), Faculdade de Odontologia de Araraquara, Departamento de Dentística Restauradora, Araraquara, SP, Brasil; 2 University of Toronto, Faculty of Dentistry, Department of Clinical Sciences - Restorative, Toronto, Canada; 3 University of Toronto, Faculty of Dentistry, Department of Basic Sciences, Toronto, Canada

**Keywords:** Tooth bleaching, Whitening toothpaste, Optical illusions, Activated charcoal

## Abstract

The efficacy of whitening toothpastes is questionable and controversial. Clinicians, patients and researchers have expressed concern with whitening toothpastes due to the risk of wearing the dental structure and the potential for disappointment if the advertised cosmetic results are not achieved. Objective: This study compared the whitening performance of toothpastes with different whitening technologies after initial and continued use. Material and Methods: Ninety bovine incisors were stained using a concentrated solution of black tea. They were randomly distributed into 6 groups, according to the toothpaste whitening technology: activated charcoal (B&W), blue covarine (WAD), hydrogen peroxide (LWA), microbeads (Oral B 3D White Perfection – 3DW) and optimized abrasives (XW4D). They were compared to a traditional toothpaste without a whitening agent (TA – control). Specimens underwent a brushing machine with controlled pressure, time and temperature. A calibrated examiner measured the color using a VITA-Classical scale before the first brushing cycle (T0), after the first brushing cycle (TI), and after a brushing cycle that simulates continuous use (TCU). Whitening performance was evaluated by the difference of shades (ΔSGU) between T0–TI and T0–TCU timepoints, using the Kruskal-Wallis and Dunn's non-parametric test. The Wilcoxon test was used to evaluate the cumulative effect (α=0.05). Results: Statistically significant differences were observed between toothpastes in both TI and TCU (p<0.05). The time of use also had a significant effect (p<0.05). Conclusion: Only WAD and 3DW showed whitening performance after the first use (TI). The greatest whitening performance after continuous use was obtained by WAD, followed by LWA and 3DW. The use of conventional toothpaste (TA) promotes no tooth whitening. Clinical relevance: Microbead abrasives (3DW) and blue covarine (WAD) were the active technology tested that presented the best global tooth whitening performance.

## Introduction

Despite unparalleled results, tooth whitening with concentrated peroxides results in high rates of side effects and serious biological risks.^1^ ^,^ ^2^ These side effects have led to increasingly restrictive regulations concerning the use of whitening products containing high concentrations of peroxide and even questions regarding the use of the procedure at all.^3^ ^,^ ^4^ Alternatively, aesthetic improvements can also be achieved with whitening toothpastes.^5^ These toothpastes offer the same therapeutic benefits (anti-caries and anti-gingivitis) of conventional toothpastes with added whitening activity from abrasives, adsorbent particles, peroxides, enzymes or optical effect agents.^6^ ^,^ ^7^

Whitening toothpaste with formulations containing hydrated silica, calcium carbonate, dicalcium phosphate dihydrate, calcium pyrophosphate, alumina, perlite or sodium bicarbonate mechanically remove pigmented biofilms and chromophores on the surface of the dental enamel. In addition, the daily use of these abrasives modifies the enamel surface, reducing the adhesion of dental biofilms and chromophores, reducing tooth pigmentation and altering its coloration.^7^ ^,^ ^8^ Whitening toothpastes containing oxidants or enzymes chemically modify pigments adhered to the teeth, reducing the intensity and appearance of discoloration.^6^ ^,^ ^7^ Optical modifying toothpastes contain pigments such as blue covarine, which act to shift the apparent color of teeth by depositing a thin, semitransparent film of bluish pigment on the dental surface. This film instantly modifies the interaction of incident light resulting in teeth that appear whiter and brighter.^6^ ^,^ ^7^ ^,^ ^9^ ^,^ ^10^ Recently, activated charcoal/carbon has attracted interest because it acts in a high surface area and consequently has the capacity of adsorbing pigments, chromophores and stains responsible for the color change of teeth. Several whitening toothpastes now incorporate activated charcoal/carbon in their formulations.^11^ However, to our knowledge, no prior studies have evaluated its effectiveness yet.

A recent systematic review demonstrated the effectiveness of whitening toothpastes compared to conventional toothpastes,^5^ but there is a lack of comparative studies to determine which of these technologies is the most effective at whitening teeth. Moreover, there are serious doubts about the effectiveness of toothpastes that use pigments of optical effect. While Collins, et al.^9^ (2008) and Joiner, et al.^10^ (2008) have demonstrated immediate, perceptible results that persist for up to 8 hours after a single application of the product, Torres, et al.^12^ (2013), Dantas. et al.^13^ (2015), Oliveira, et al.^14^ (2016), Horn, et al.^15^ (2014) and Bortolatto, et al.^16^ (2016) have failed to demonstrate whitening effects superior to that promoted by conventional toothpaste.

From a practical point of view, it is important that the whitening performance of these toothpastes is visibly perceptible to patients and professionals, because the visual perception under daily conditions is how the effectiveness of products is judged. It is important to analyze the efficacy of these toothpastes not only by standard laboratory methods, but by visual comparative methods, even if these methods are less reducible and more subjective than instrumental methods with spectrophotometers and colorimeters.^17^

The establishment of tooth whitening performance of each of these technologies will contribute to a better understanding of the advantages and limitations of each whitening toothpaste tested. Furthermore, it will provide both patients and dental professionals the ability to critically select a product that is the best fit for them, based on scientific results and without the risk of being misled by commercial advertisements promising unattainable results.^18^ This study evaluates the whitening performance after both first and continuous use of different whitening toothpastes. The null hypothesis is that there is no difference in the whitening performance between different whitening toothpastes both after the first application and after the continuous use of the toothpastes.

## Material and methods

This *in vitro* study was approved by the Animal Ethics Committee of the Araraquara School of Dentistry – UNESP, São Paulo, Brazil (protocol number: 25/2014).

### Study design

**Figure 1:**
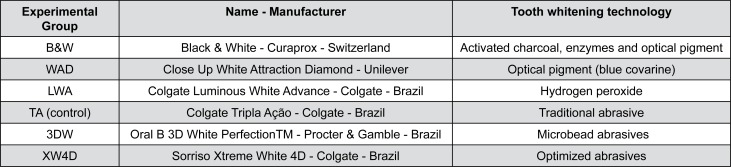
Experimental group, name, manufacturer and the type of tooth whitening technology used in each toothpaste analyzed in this study

Source: https://www.curaprox.com/data/downloads/21427792206Black_Is_White_e.pdfhttp://www.closeup.com.br/product/detail/512432/white-attraction-diamondhttp://www.colgate.com.br/app/Luminous/Equity/BR/Produtos/Cremes-Dentais/colgate-luminous-white-advanced.cvsphttp://www.colgate.com.br/pt/br/oc/products/toothpaste/colgate-tripla-acao-hortelahttps://oralb.com.br/pt-br/produtos/creme-dental-oral-b-3d-white-perfectionhttp://www.colgate.com.br/app/PDP/Sorriso/BR/produtos/cremes-dentais/sorriso-xtreme-white-4d.cvsp

This randomized, controlled, double blinded, *in vitro* study was conducted to compare the whitening performance after the first use and after continuous use of whitening toothpaste. The whitening toothpastes used in this study were grouped by their active whitening technology: activated charcoal, hydrogen peroxide, optical dye or microbead abrasives (Figure 1). The control group was Colgate Triple Action toothpaste, which has no whitening component in its formulation.

### Calibration of the examiner

The examiner (DJ) responsible for recording the color of each tooth was trained to use a Vita Classical shade guide ordered by value (VITA Zahnfabrik, Bad Säckingen, BW, Germany).^19^ ^,^ ^20^ The shades of 20 non-stained bovine incisors were recorded at two distinct times with a 7-day gap between evaluations. This data was scored and the level of intra-examiner reproducibility was determined by the Kappa coefficient. The examiner was considered calibrated after a Kappa coefficient = 0.647, which is considered a substantial agreement.^21^

### Blinding

Both the examiner responsible for recording the tooth color and the researcher responsible for performing the statistical analysis were blinded for the toothpaste used in each experimental group. Specimens were numbered and randomly distributed in the experimental groups according to a randomly generated list obtained from the RANDOM.ORG. Specimens were assembled on a silicone block, which immobilized them on the brushing machine. The sequence of toothpaste application was randomized with the same method.

### Sample size calculation

The software G*Power NT was used to determine the minimum sample size according to the following parameters: 95% statistical significance, 0.80 test power, 0.40 effect size and 6 experimental groups. This resulted in a minimum sample size of 90 specimens (N=90).

### Sample preparation

**Figure 2:**
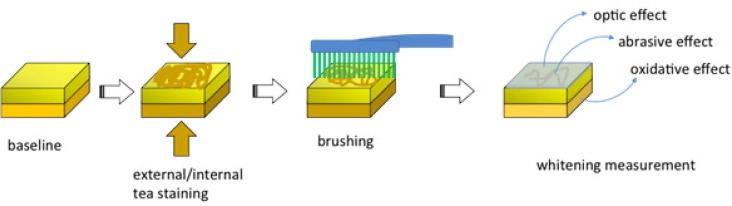
Staining protocol of the sample and possible whitening effects of the different whitening toothpastes tested, as a function of their whitening technology

The calibrated examiner inspected 300 bovine incisors and selected 90 teeth from this set. Selection criteria for this study was: similar shade, size, and surface texture. First, the roots of the 90 incisors were removed. Then, the pulp chamber was accessed and extended using a conical diamond tip (3147 KG Sorensen). Afterwards, the dental pulp and debris were removed with the aid of dental curette and air/water jets until the pulp chamber was completely empty. Subsequently, the specimens were immersed in a concentrated solution of black tea prepared by the infusion of 500 mL of water and 16 g of tea (10 sachets). The staining protocol consisted of 18 hours of immersion in the tea infusion followed by 6 hours of drying at room temperature. All teeth were submitted to 4 complete cycles of staining. After staining, the pulp chambers were filled with red acrylic resin (Resinlay – Dencor, Artigos Odontológicos Clássicos Ltda, São Paulo, SP, Brazil) to simulate the natural coloration of dental pulp. Figure 2 summarizes external and internal staining protocols as well as whitening effects of the different technologies tested in this study.

After staining, all specimens had a Vita A4 shade. These were stored for 7 days in artificial saliva. After this period, the initial color was recorded before toothpaste application (T0).

### Brushing protocol

Specimens from each treatment were randomly grouped in a 3×3 orientation and fixed with a condensation cured silicone rubber impression material (Zetaplus – Zermack, Badia Polesine, RO, Italy). They were attached to a brushing machine (MEV 2T – ODEME, Luzerna, SC, Brazil) with the vestibular face exposed to the external environment. Each specimen was submitted to two brushing cycles. The first cycle consisted of 180 toothbrush head movements (first use – TI). The second one, with 16,200 movements, was performed to simulate a continuous use (TCU). All specimens were brushed with a soft bristled toothbrush (Colgate Classic – Colgate, São Paulo, SP, Brazil). Both the pressure (200 g/cm^2^) and temperature (36±1°C) were controlled. During brushing, samples were immersed in a solution of toothpaste, artificial saliva and distilled water obtained by dilution of equal volumes of each component (1:1:1). After each brushing cycle, specimens were washed in running water and the color was measured immediately after washing.

### Whitening efficacy

A previously calibrated examiner compared the color of each specimen using the Vita Classical shade guide, which consists of 16 tabs arranged from highest (B1) to lowest (C4) value.^22^ ^–^ ^25^ The color was recorded before any toothpaste application (T0) and after both TI and TCU.

These evaluations were held inside a metamerism chamber (Color Viewing Booth® – metameric system – Mako, Rio Negro, PR, Brazil) under standard conditions at a distance of 30 cm, capture angle of 45° and daylight illumination (illuminant D65, daylight with 6504K color temperature). The recorded tones were converted into scores and the whitening efficacy into shade guide units (ΔSGU) was determined by the difference between the scores recorded before (T0) and after the brushing cycles (TI and TCU) according to Equation 1 19, 20:

$$\left(1\right)\Delta SGU=\left({\text{score}}_{T0}-{\text{score}}_{T\text{I}}\text{or}{\;}_{TCU}\right)$$

### Statistical analysis

In this study, the median was used as measure of central tendency, due to the type of data (color shade score) and primary outcome (whitening efficacy). The non-parametric Kruskal-Wallis (KW) test was used to analyze the whitening efficacy at each time analyzed (TI and TCU), supplemented by Dunn's multiple comparison test. The effect of long-term toothpaste use on the whitening efficacy was analyzed by the non-parametric Wilcoxon (W) test of repeated measures. All statistical analyses were performed by IBM SPSS Statistics 19 software (IBM SPSS Statistics, Chicago, IL, United States of America), considering a 95% statistical significance.

## Results

The experimental sample consisted of 90 bovine incisors stained with black tea with an average initial A4 color shade on the Vita Classical shade guide (SGU=15.11); standard deviation of ±1.33, 0.088 coefficient of variation and 15.0 median at baseline (T0).

**Figure 3:**
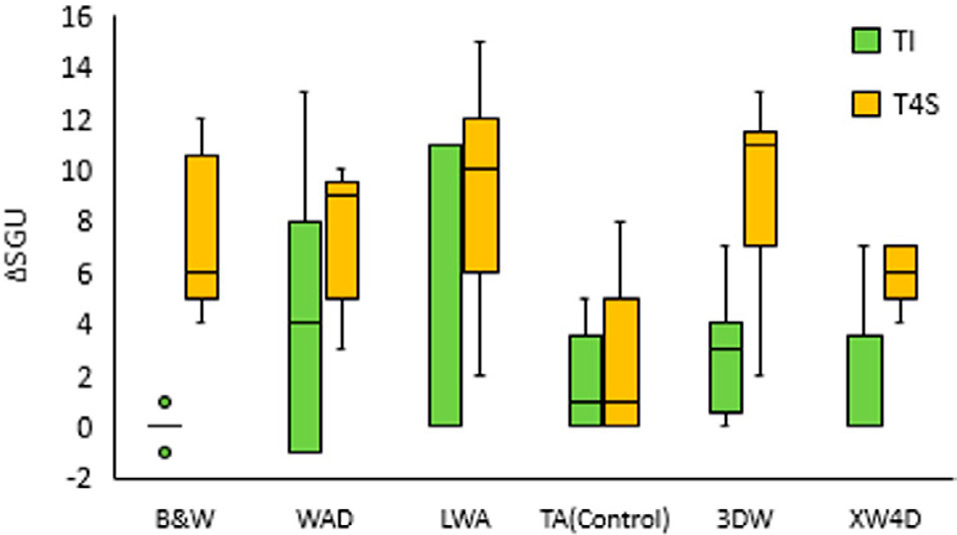
Whitening efficacy values expressed by the difference in shades units (ΔSGU) between the baseline (T0) and after first use (TI) and after 4 weeks of use (T4S) for each toothpaste

Table 1 shows the values for whitening performance after the two periods evaluated. Statistically significant differences were observed between the ΔSGU values after the first use (X^2^_KWTI_(5)=16.351; p=0.0001; N=90), and also after the continuous use (X^2^_KWTCU_(5)=17.857; p=0.0001; N=90). The statistically significant differences (p<0.05) in the whitening performance at each time are highlighted in Figure 3.

**Table 1:** Whitening efficacy values expressed by the difference of shades (ΔSGU) between baseline (T0), after first use (TI), and continuous use (TCU) of toothpaste

Time of use	Toothpaste	Mean	Standard deviation	Coefficient of variation	Median
TI	B&W	0.00	±0.54	0.00	0.00
	WAD	4.60	±5.08	1.10	4.00
	LWA	4.40	±5.58	1.27	0.00
	TA (control)	1.60	±1.99	1.25	1.00
	3DW	2.67	±2.47	0.92	3.00
	XW4D	1.87	±3.20	1.71	0.00
TCU	B&W	7.40	±3.14	0.42	6.00
	WAD	7.20	±2.73	0.38	9.00
	LWA	9.00	±4.72	0.52	10.00
	TA (control)	2.47	±2.75	1.11	1.00
	3DW	8.80	±3.57	0.41	11.00
	XW4D	5.93	±1.22	0.21	6.00

The repeated measures Wilcoxon test showed a statistically significant increase in whitening (Z=-7.164; pUE=0.001; N=90), due to the continuous use (xΔSGUTI=2.52 and xΔSGUTCU=6.80) for all whitening toothpastes. However, the control toothpaste (TA) showed no significant whitening change (Figure 3).

## Discussion

This study showed a variety of technologies used in whitening toothpastes and found different whitening efficacies compared to the control and between the different groups. The null hypothesis was rejected because statistically significant differences were observed between and within groups. One class of whitening toothpastes relies on mechanical or abrasive activity to remove biofilms and chromophores adhered to the dental enamel surface, improving the aesthetics by restoring the natural dental color.^8^ ^,^ ^17^ In addition, the continued use of more abrasive toothpastes can increase brightness and reflectance of the enamel, resulting in a more pleasing appearance of whiter and brighter teeth.^17^ However, the possible early and excessive wear of dental enamel is a matter of concern, especially when whitening toothpastes are used by children or individuals with acid erosion and/or abfraction lesions^17^ ^,^ ^26^ This has motivated the development of alternative technologies that allow teeth whitening without the risk of causing damage to the dentition.

The use of blue covarine and other pigments of optical effect, such as FD&C Blue No. 1, in whitening toothpastes modifies the perception of yellowish discoloration in teeth by depositing a thin, semi-transparent blue layer on the dental enamel. Blue opposes yellow in the color spectrum, creating the visual appearance of whiter and brighter teeth by shifting the net color towards white. This mechanism of whitening action is based on the study of Kleber, et al.^27^ (1998) and Gerlach, et al.^28^ ^,^ ^29^ (2000,2002), in which they demonstrated that the bluish shift of the yellow-blue axis (b*) is more relevant for the perception of whiter and brighter teeth than an increase in luminosity (L*) or changes in the green-red axis (a*) within the CIELab color space. The literature provides conflicting reports concerning the effectiveness of pigments of optical effect in tooth whitening. Collins, et al.^9^ (2008), Joiner, et al.^10^ (2008), Tao, et al.^30^ (2017), Tao, et al.^31^ (2017) and Bergesch, et al.^32^ (2017) showed immediate and progressive efficacy; however, studies by Torres, et al.^12^ (2013), Horn, et al.^15^ (2014) and by the present research group reported no whitening effect of these compounds.^13^ ^,^ ^14^ ^,^ ^16^

Analyzing the contradictory results in literature, this study found that the research confirming the whitening effect of toothpastes containing blue covarine had quantified the tooth color by visual comparison. This includes the use of Vita Classical (ΔSGU), Vitapan 3DMaster 30 and other scales; or luminosity difference (ΔL*), variation of the red-green (Δa*) and blue-yellow (Δb*) axis, and the objective whitening index (WIO), calculated from the CIELab color space coordinates obtained with the chromameter Minolta CR241;^10^ ^,^ ^30^ ^,^ ^31^ or digital photographs analyzed in Adobe Photoshop,^9^ ^,^ ^32^ while studies that did not prove the whitening effect measured the tooth color with the Vita EasyShade reflectance spectrophotometer.^12^ ^–^ ^16^ Although this instrument is effective and reliable for studying teeth whitened with peroxides,^20^ ^,^ ^33^ it does not appear to be suitable for recording the surface optical effect of blue covarine. The explanation for this limitation was found only in a technical document published by the manufacturer #20030915-1^34^, stating that the Vita Easyshade is specially designed to ignore the reflection of enamel and record only the scattered light reflected by the dentin. Based on this, and considering the articles by Meireles, et al.^19^ (2008) and Mena-Serrano, et al.^20^ (2016), in the present study the whitening efficacy was analyzed only by visual comparison using the Vita Classical scale.

In this study, TI was used to test the immediate whitening effect induced by toothpastes containing blue covarine. WAD (M_eΔSGUTI=4.00) and 3DW (M_eΔSGUTI=3.00) were the only formulations that showed effective whitening after a single application. These results corroborate the findings of Collins, et al.^9^ (2008), Joiner, et al.^10^ (2008), Tao, et al.^30^ (2017), Tao, et al.^31^ (2017) and Bergesch, et al.^32^ (2017), who also observed the efficacy of agents of optical effect as alternative technologies for tooth whitening. However, despite containing an optical pigment in its formulation, LWA surprisingly exhibited no comparable effect to the other toothpaste of optical effect. The lack of immediate efficacy of this toothpaste is probably due to the low concentration of pigment of optical effect in its formulation, since Tao, et al.^30^ (2017) stated that the whitening efficacy of blue covarine is directly proportional to its concentration in the toothpaste. Another explanation could be that the other components (activated charcoal and enzymes) neutralize the action of this optical agent. Activated charcoal is especially adept at pigment removal, supporting this explanation.^35^ ^–^ ^37^

The whitening effect of activated charcoal is based on its high capacity to adsorb and retain chromophores in the oral cavity. Activated charcoal is highly porous and has an extremely high surface area,^11^ resulting in effective and progressive cleaning of the dentition. Despite Brooks, et al.^11^ (2017) reporting that this possible whitening agent has no scientific proof, 96% of commercially available activated charcoal toothpastes claim to effectively whiten teeth in their marketing materials.

A search for scientific articles published in English from January 2018 was performed in PubMed MEDLINE and Scopus databases to explain this whitening effect. The search terms used were: 1) charcoal AND tooth OR dental AND whitening OR bleaching, found 1 Scopus and 0 Pubmed; and 2) charcoal AND toothpaste OR dentifrice; no scientific papers evaluating the effect of this technology for tooth whitening were found. Therefore, this study is believed to be the first evaluating the whitening efficacy of activated charcoal toothpaste, which had no immediate effect (p>0.05), as observed for toothpastes WAD and 3DW.

After continuous use, all whitening toothpastes had a statistically different effect and were superior to the control treatment (TA). The better technologies of whitening toothpastes were microbeads in 3DW (M_e_ΔSGU_TCU_=11.00), hydrogen peroxide in LWA (M_e_ΔSGU_TCU_=10.00) and blue covarine in WAD (M_e_ΔSGU_TCU_=9.00). Effective whitening was also demonstrated by B&W activated charcoal (M_e_ΔSGU_TCU_=6.00) and XW4D optimized abrasives (M_e_ΔSGU_TCU_=6.00); however, the whitening was statistically lower than with LWA, 3DW and WAD. Conventional toothpaste (TA) showed no whitening efficacy even after 4 weeks of consecutive use. The most effective product over both time periods was 3DW.

In spite of new technologies, such as blue covarine and microbeads, having the advantage of an immediate whitening effect,^9^ ^,^ ^10^ ^,^ ^30^ ^,^ ^32^ this study is in agreement with van Loveren and Duckworth^7^ (2013), believing that the component that differentiates the whitening efficacy of the toothpaste is the effectiveness of the optimized abrasives present in their formulations. Questions raised by Al-Tarakemah and Darvell^18^ (2016) regarding both the biological risks of tooth whitening with peroxides and the efficacy of blue covarine toothpastes are relevant and important to practitioners and patients. This study showed that an effective aesthetic improvement can be achieved by all the tested whitening toothpastes after regular use. These results are useful to guide patients and dental professionals in the selection and use of whitening toothpaste products. In addition, this study shows that concerns about efficacy of toothpastes containing blue covarine may be a result of limitations in previous study methodologies. Instrumentation and methods used for determining tooth whitening in traditional products may incorrectly assess the whitening effect of toothpastes with optical pigments. Thus, this study demonstrated that the visual assessment method used was adequate to evaluate the whitening performance of a variety of whitening toothpastes, and was especially useful in toothpastes containing optical pigments, since the easyshade spectrophotometer was not designed to evaluate color changes on the surface of the dental enamel.

## Conclusion

The results of this *in vitro* study demonstrated that all whitening toothpastes were effective for whitening teeth when compared to a toothpaste without added whitening agents (TA). The best whitening performance was obtained with microbeads (3DW), followed by hydrogen peroxide (LWA) and blue covarine (WAD). Continuous use enhances the whitening performance of all whitening toothpastes. The low initial performance of the control toothpaste (TA) did not improve.
